# Genetic variation in neurodegenerative diseases and its accessibility in the model organism *Caenorhabditis elegans*

**DOI:** 10.1186/s40246-017-0108-4

**Published:** 2017-05-25

**Authors:** Yiru Anning Wang, Jan Edward Kammenga, Simon Crawford Harvey

**Affiliations:** 1 Biomolecular Research Group, School of Human and Life Science, Canterbury Christ Church University, Canterbury, CT1 1QU UK; 2 Laboratory of Nematology, Wageningen University, 6708 PB Wageningen, The Netherlands

**Keywords:** Neurodegenerative diseases, Natural variation, Quantitative genetics, *C. elegans*

## Abstract

**Background:**

Neurodegenerative diseases (NGDs) such as Alzheimer’s and Parkinson’s are debilitating and largely untreatable conditions strongly linked to age. The clinical, neuropathological, and genetic components of NGDs indicate that neurodegeneration is a complex trait determined by multiple genes and by the environment.

**Main body:**

The symptoms of NGDs differ among individuals due to their genetic background, and this variation affects the onset and progression of NGD and NGD-like states. Such genetic variation affects the molecular and cellular processes underlying NGDs, leading to differential clinical phenotypes. So far, we have a limited understanding of the mechanisms of individual background variation. Here, we consider how variation between genetic backgrounds affects the mechanisms of aging and proteostasis in NGD phenotypes. We discuss how the nematode *Caenorhabditis elegans* can be used to identify the role of variation between genetic backgrounds. Additionally, we review advances in *C. elegans* methods that can facilitate the identification of NGD regulators and/or networks.

**Conclusion:**

Genetic variation both in disease genes and in regulatory factors that modulate onset and progression of NGDs are incompletely understood. The nematode *C. elegans* represents a valuable system in which to address such questions.

## Background

**Fig. 1:**
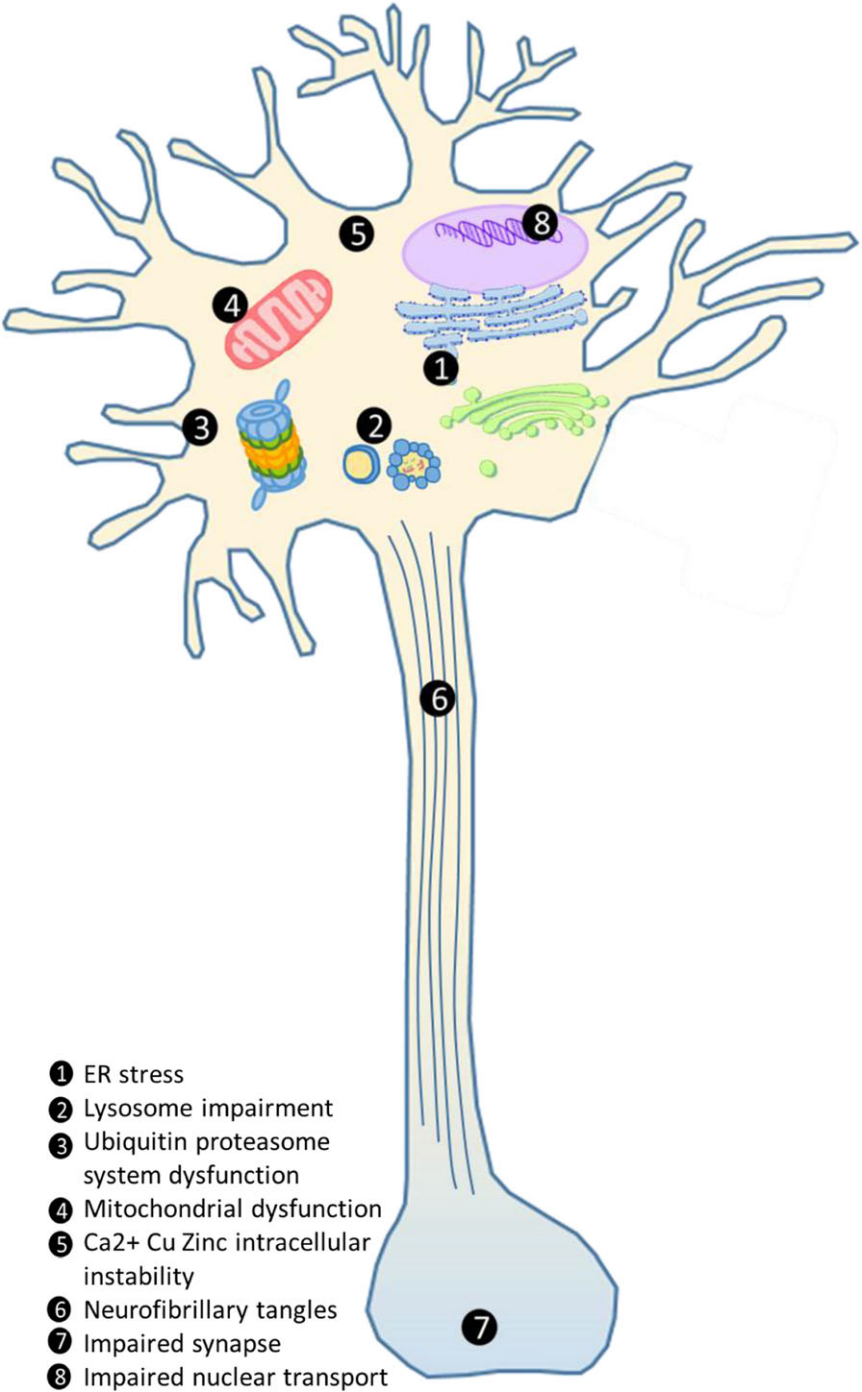
Schematic diagram of the pathological mechanisms associated with neurodegeneration in neurons. Eight main events exist in the cellular biological processes related to neurodegeneration: *1* The protein-misfolding process inhibits ER to Golgi trafficking and alters ER-associated degradation, inducing the ER stress [55]; *2* Damaged lysosomes disrupt processes that maintains lysosomal homeostasis [78]; *3* Accumulation of misfolded proteins generates positive feedback exacerbating other effects [15]; *4* Dysfunctional mitochondrial maintenance directly affects mitochondrial biogenesis and induction of autophagy [75], e.g., the production of ROS and ATP in the cell; *5* Altered homeostasis generates excessive influx of calcium, copper, and zinc, due to ER and mitochondrial stresses [78]; *6* Plaques and neurofibrillary tangles, due to tau phosphorylation and aggregates, reduce neurotransmitter release, which also *7* weaken synaptic strength [15]; *8* Signaling pathways in the stressed organelle or the cytoplasm induce the transductions of the signals to the nucleus, which provoke DNA damage [10, 80]

Neurodegenerative diseases (NGDs) cause disability and premature death, primarily among older people. These chronic and fatal illnesses include Alzheimer’s disease (AD), Parkinson’s disease (PD), Huntington’s diseases (HD), spinocerebellar ataxia, prion diseases (PrD) (i.e., transmissible spongiform encephalopathies), frontotemporal dementia (FTD), and amyotrophic lateral sclerosis (ALS) [88]. Most NGDs are age dependent with their incidence increasing with advancing age. This makes understanding NGDs increasingly important given the recent increase in human lifespan seen in many countries. Many disease-causing mutations underlying different NGDs have phenotypic effects that result from the misfolding of proteins and/or mitochondrial dysfunctions leading to widespread damage in different parts of the nervous system (Fig. 1). This damage leads to a range of symptoms, and the overlap between symptoms in different NGDs can make it difficult to precisely diagnose patients. Diagnosis is further complicated by the individual variability apparent from the onset of disease [99]. For example, PD is characterized by motor issues, including tremor, slowing of movement, and an unstable gait, and by cognitive symptoms, but only some patients suffer from cognitive impairment and develop dementia [20].

The rate of disease progression (i.e., the duration of a given neuropathological stage) and clinical presentation also vary from one patient to another. Young onset PD patients, for example, often have a more frequent family history of PD and a more variable survival rate relative to those without the familial history [79]. Several studies of the amyloid-β protein aggregates, which cause AD, also indicate that the existence of distinct shapes in beta amyloid peptide (Aβ) aggregates, 40 residue Aβ (Aβ40) and 42 residue Aβ fibril structures [51], and identify the distinct strain-specific traits (defined as “strainness”) of the forms of AD by the different conformation of the aggregates [91, 104].

There is however limited knowledge about the mechanisms that determine individual variation. Individuals that carry the same mutation in the same disease-causing gene may display a range of different clinical symptoms. For example, assessing 6-year change in verbal memory, processing speed, and executive function in AD identified effects of MS4A4E, CLU, and NME8 in whites and of ZCWPW1 and CDS33 variants in African Americans. For MS4A4E and CLU, this association was only significant in individuals bearing at least one *APOE ϵ4* allele (AD risk gene) [9]. Apart from lifestyle and environment, these individual differences are caused by the unique genetic background of each person. The genetic background, thus, could be defined as the genetic makeup of all alleles that interact with the disease related to the “disease-causing” mutation.

Detailed mechanistic studies into background modifiers of NGDs are however difficult to conduct at the individual level in humans for ethical and technical reasons. Model organisms such as yeast, insects, worms, fish, and rodents are therefore critical to furthering our understanding of differences between individual in NGDs. This use is facilitated by the range of methods and approaches available to construct transgenic models of human diseases in model species with different genetic backgrounds [1, 19, 32, 53]. They are likewise an important resource for investigating the genetic underpinnings of quantitative traits, including complex disease phenotypes. Here, we review the onset and progression of NGDs in the context of genetic background and illustrate how work on the model species *Caenorhabditis elegans* can illuminate the underlying mechanisms of individual variation.

### NGD phenotypes depend on the genetic background

Individual genetic backgrounds differ from thousands to millions of genetic variants that will range from single-nucleotide polymorphisms (SNPs) to, potentially very large, copy number variants (CNVs) [92, 109]. This genetic variation is a major determinate of differences in predisposition to disease [1, 41], where risk-increasing variants are numerous, display intricate patterns of interaction with each other as well as with non-genetic variables, and unlike classical Mendelian (“monogenic”) disorders will often exhibit no simple mode of inheritance. Differential phenotypes for most NGDs arise from multiple genetic variants and their interaction with each other, as well as environmental factors. Hence, the genetics of these diseases is considered “complex” based on the heterogeneity in pathology and the disease polygenicity [8].

Genetic background can affect an important feature shared by different NGDs, specifically the formation of cerebral deposits of misfolded protein aggregates (also called prion-like proteins). For example, AD is characterized by the presence of neurofibrillary tangles and beta amyloid peptide (Aβ) in neural plaques, which are abnormal accumulations of microtubule-associated protein tau in a hyperphosphorylated state [82]. Similarly, the accumulation of proteins with polyglutamine-rich extensions is characteristic of HD and associated polyglutamine diseases [13], while PD involves the loss of dopaminergic neurons and the presence of Lewy bodies and Lewy neurites that are the aggregates of the synaptic protein alpha-synuclein. However, exactly how genetic variation modifies and affects specific parts of the NGD pathways is mostly unclear. We suggest that the genetic variation associated with NGDs can be grouped into two classes. Firstly, variation present directly in disease genes. Secondly, variation in regulatory factors that modulate onset and progression of the NGDs. Critically, this distinction separates those variations that are causative of disease from those that modify the disease but that on their own, cannot cause NGD.

**Fig. 2:**
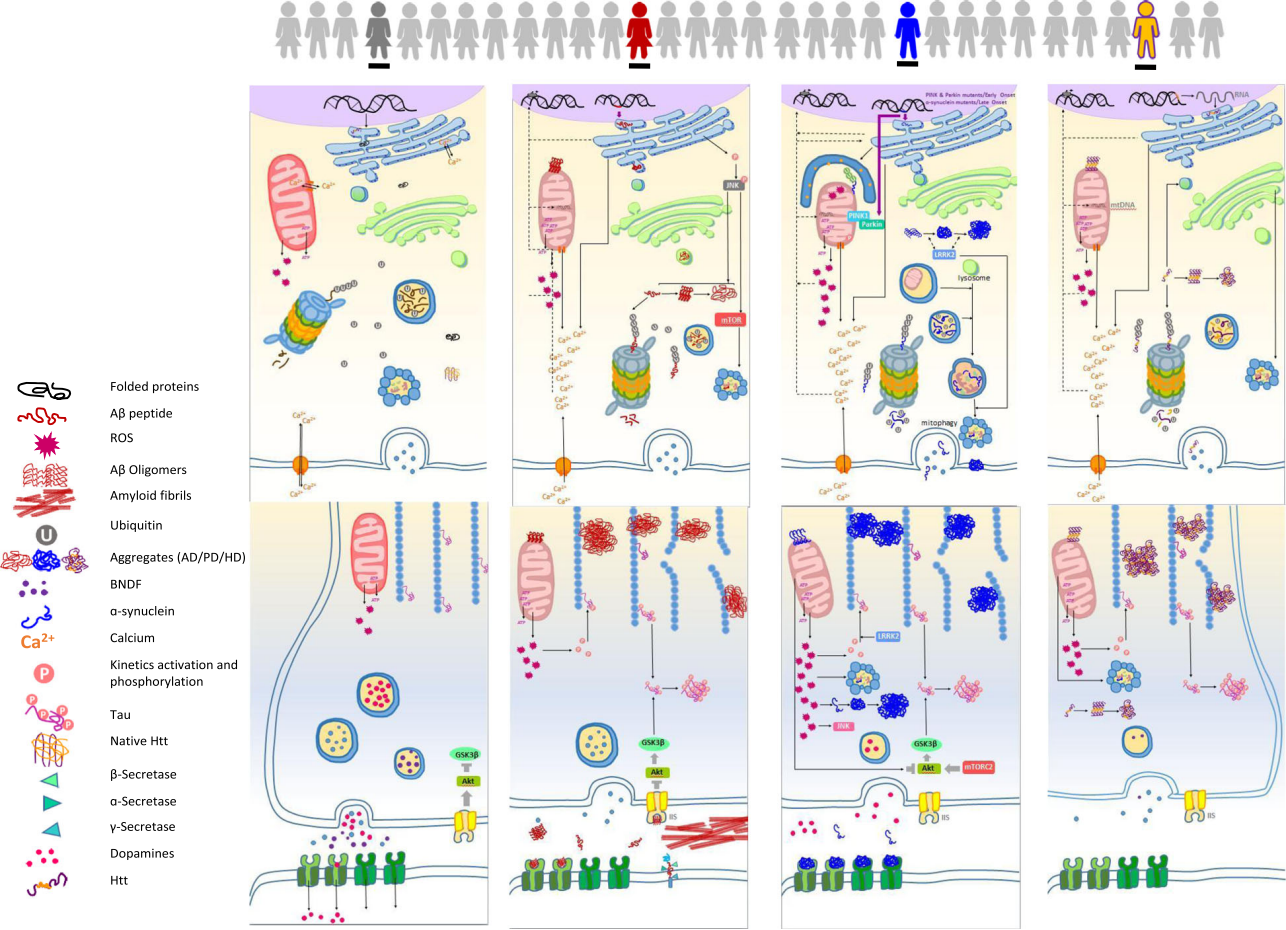
The molecular processes implicated in neurodegeneration in the neurons of a normal healthy individual (*gray*) and in AD (*red*), PD (*blue*), and HD (*yellow*) patients. The schematic neuron is divided into the soma and axonal terminal bouton. For simplicity, postsynaptic/dendritic events are not included. Misfolded proteins aggregate first into oligomers and then into higher-molecular-weight insoluble protofibrils and further aggregates [17, 37, 104]. In AD patients with mutations in APP, PSEN1, PSEN2, or APOE, the JNK pathway is activated, increasing levels of phospho-JNK in neurons. This mediates the phosphorylation of APP and FoxO-dependent autophagy [52, 63]. Moreover, the soluble Aβ oligomers activate the mTOR pathways again promoting autophagy [52]. Further phosphorylation of tau and impaired Aβ activate the IIS/Akt pathways and affect cognitive function and synaptic plasticity [38]. In PD, mutations in PINK and Parkin (related to EOPD) or α-synuclein mutations (LOPD) lead to the inhibition of α-synuclein degradation as well as accumulation of autophagic vacuoles, which result in neuronal death [16, 96]. Misfolded α-synuclein also interacts with membranes and mitochondria, causing calcium dysregulation and a reduction of mitochondrial activity. This results in mtDNA damage as well as impairments to the ubiquitin-proteasome system (UPS) and mitophagy. The significant pathological etiology of HD is the enlargement of the polyglutamine (polyQ) domain within the HTT protein’s N-terminus [110]. MLH1 (MutL homolog 1) and an SNP within a nuclear factor-κB binding site (Nf-KB) in the HTT promoter play a role in the altered onset of HD. In comparison with AD and PD, proteasome efficiency is strongly reduced in HD patients. Meanwhile, the polyQ domain of mutant HTT contributes increased toxicity by attracting and binding to other cytoplasmic and nuclear structures that contain polyglutamine (reviewed by [6]). Additionally, a major loss of brain-derived neurotrophic factor (BDNF) protein has been shown in HD and may due to the deficits in BDNF delivery and/or loss of BDNF gene transcription by mutant Htt [23, 39]

**Fig. 3:**
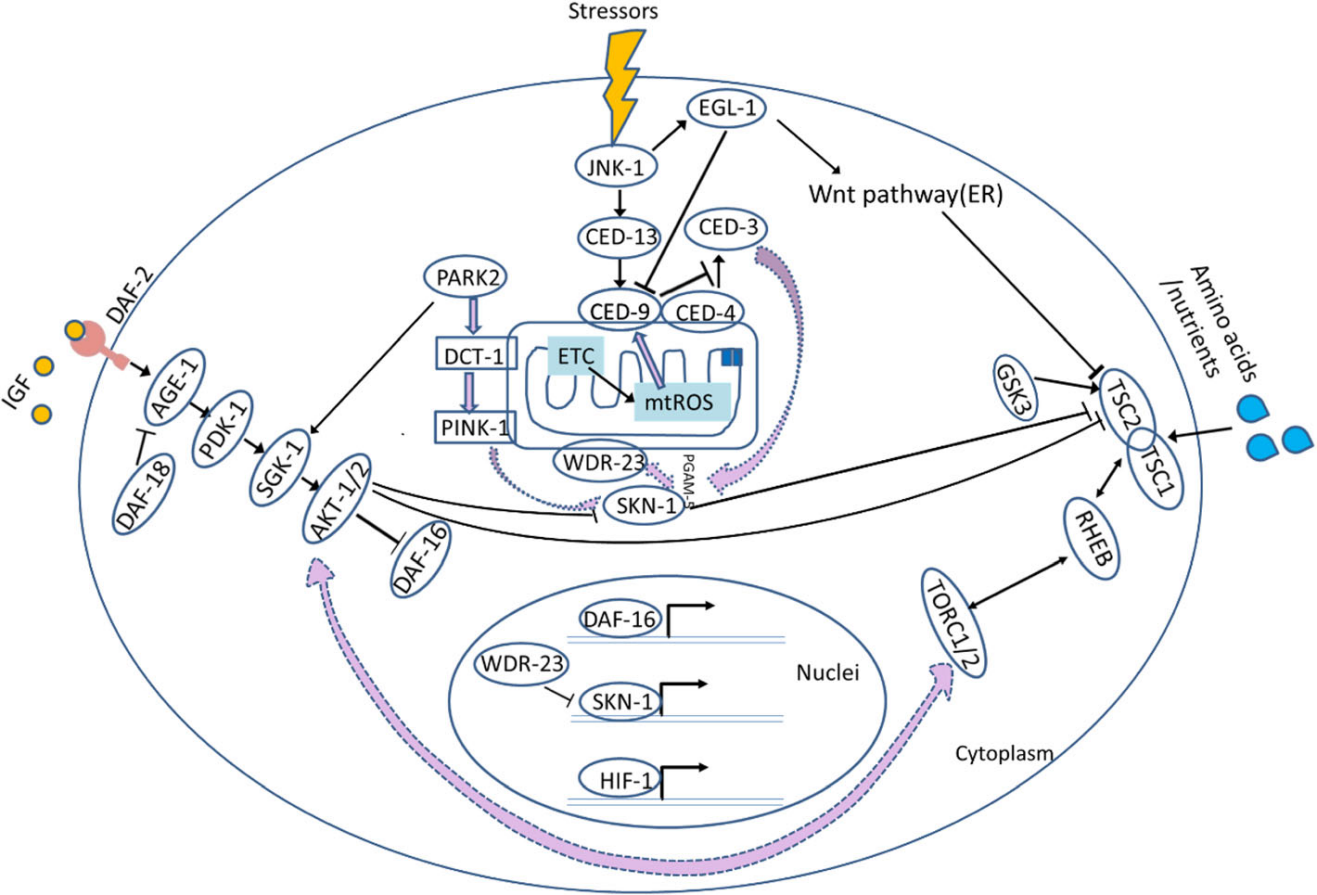
*C. elegans* cellular pathways and their crosstalk, relating to aging and the stress response. Shown (from *left to right*) are the insulin/insulin-like growth factor (IIS) signaling pathway, the mitochondrial signaling pathways, and the mechanistic target of rapamycin (TOR) pathway. *Arrows* indicate positive regulatory events and *bars* indicate inhibitory interactions. *Purple block arrows* represent interactions between the different pathways, whereas *dashed purple block arrows* indicate possible indirect interactions. The oval molecules and their corresponding mammalian homologs involved in IIS [28, 56, 62, 72] are as below: AGE-1/PI3K, phospatidylinositol-3-kinase; PDK-1, 3-phosphoinositide-dependent kinase 1 ortholog; SGK-1, a serine/threonine protein kinase that is orthologous to the mammalian serum- and glucocorticoid-inducible kinases (SGKs); Akt/PKB, the serine/threonine kinase; DAF-16/FOXO, forkhead box O (FOXO) transcription factor. Mitochondrial dysfunctions [90–96] are associated with apoptotic/programmed cell death (PCD), aberrant autophagic regulation, endoplasmic reticulum dysfunction, and intracellular calcium, including c-Jun N-terminal kinase (JNK) subgroup of mitogen-activated protein (MAP) kinases; CED-4, CED-9, and EGL-1 belong to a conserved genetic pathway to regulate apoptosis during *C. elegans* development [63]; PINK-1, a predicted serine/threonine kinase which is similar with human *PINK1*, PTEN-induced kinase-1; *SKN-1/Nrf*, skin in excess transcription factor 1/NF-E2-related factor; *mtROS*, mitochondrial reactive oxygen species; *ATP*, adenosine-5′-triphosphate; *HIF-1*, hypoxia-inducible transcription factor 1. Major molecules in TOR pathways include *TSC1/2*, tuberous sclerosis complexes 1 and 2; *RHEB*, Ras homolog enriched in brain; *TOR*, target of rapamycin kinase; *GSK3*, glycogen synthase kinase ortholog. See text in Table 1 for further details

In an example of the first such class of variants, multiple rare mutations in amyloid precursor protein (APP), or in the presenilin-1 and 2 genes (*PSEN1* and *PSEN2*), can cause early-onset AD (Fig. 3). Similarly, multiple mutations in leucine-rich repeat kinase 2 (*LRRK2*), a gene associated with PD, are known to be related to the sporadic late-onset form of the disease. Here, the G2385R and R1628P *LRRK2* variants are validated risk factors for PD in Asian populations, while the G2019S variant has been identified in different populations worldwide [57]. Examples of the second class of variants are those known to act in the IIS/mTOR pathway (Figs. 2 and 3). This pathway modulates response to a range of stresses and in the NGDs has been linked to a range of pathological processes (Fig. 1 and Table 1). For instance, Baleriola et al. [4] reported moderate eIF2α activation by Aβ and a greater frequency of ATF4 (the eIF2α effector) transcripts were identified in axons in the brain of AD patients. ATF4 is also known to activate the Parkin gene in PD and also to be related to stress pathways [55]. ATF4 therefore induces transcriptional expression of genes mediated by the UPR, including genes involved in amino acid metabolism, resistance to oxidative stress, and the proapoptotic transcription factor *CHOP*, which are related to disease processing [4, 24]. However, rare coding variation in ATF4 has been also found with pathway impairment in patients with sporadic cervical dystonia [69]. Additionally, Chaudhry et al. [11], using gene-based analyses for late-onset AD, revealed associations with the WT1, ZC3H12C, DLGAP2, and GPR1 genes, suggesting a possible role in AD pathogenesis.

**Table 1:** Pathways regulating longevity, stress, and disease responses

*Insulin/Insulin-like growth factor (IIS) signaling pathway*. The insulin-like receptor *DAF-2* is one of the main molecular components of IIS pathway in *C. elegans*, which is activated by binding insulin-like peptides (IPs) [44]. *DAF-2* activation by IPs results in recruitment and activation of AKT and SGK-1 (which are activated by phosphorylation of PDK-1 at the plasma membrane) to phosphorylate and sequester DAF-16 in the cytosol [95]. When activated by stresses, dephosphorylated DAF-16 enter the nucleus and regulates the transcription of a large number of genes involved in resistance to abiotic and biotic stresses, dauer larvae formation, metabolism, and longevity [63, 98]. DAF-16 can become dephosphorylated by the absence of the IP ligand(s), by inhibition from upstream pathway members—e.g., DAF-18 opposes the activity of PDK-1 and AKT-1 via inhibition of AGE-1—or by mutations in upstream genes, leading to lifespan extension [28, 56, 62, 72]. Strikingly, downregulation of IIS via *daf-2* or *age-1* mutations in *C. elegans* delays the formation of small aggregates in nematode models of NGDs and also slows the onset and decreases the severity of associated pathologies in a *daf-16*-dependent manner [17].
*Mechanistic target of rapamycin (mTOR) pathway*. The kinase mTOR integrates nutrient and anabolic signals to promote growth [52, 106, 112]. The TSC1/TSC2 complex acts as a negative regulator of mTORCs upstream, RHEB, but can be activated by AKT kinase (which belongs to IIS pathway) [36]. The mTORC signaling regulates a large number of developmental processes. TORC1 signaling transactivates/represses PH4-4 that induces pro-survival factors expression for life extension under nutrient restriction [85]. Moreover, TORC1 can increase protein synthesis and extends lifespan by both activating the ribosomal subunit S6 kinase (S6K) and inhibiting 4E-BP1 (a negative regulator of translation) [81, 97]. Meanwhile, investigations in *C. elegans* as well as other organisms have shown that autophagy is induced by inhibition of TORC1 [46]. An inadequate level of autophagy has been implicated in physiological responses to exercise and aging as well as in pathophysiological processes, such as cancer and metabolic and neurodegenerative disorders [47]. Although mammalian TORC2 (mTORC2) signaling shows insensitivity to nutrients in comparison to mTORC1, it does respond to growth factors like insulin through a poorly defined mechanism that requires PI3K (the *C. elegans* homolog is AGE-1) [111]. Moreover, mTORC2 directly activates AKT and SCK-1 which regulate cellular processes such as metabolism, survival, apoptosis, growth, and proliferation through the phosphorylation of several effectors [47]. In addition, TORC2 controls the cell cycle-dependent polarization of the actin cytoskeleton, which is involved in complex liposome mechanism, inducing several processes required for cancer cell growth, survival, and proliferation. Functioning as a nutrient sensor—detecting nutrients and amino acids—mTOR has a complex influence on several crucial cellular functions and shows clear effects on aging, protein synthesis and autophagy, and the homeostasis pathways that play a key role in the mechanisms that affect NGDs.
*Mitochondrial signaling pathway*. Mitochondria play important roles in aging and disease through apoptotic/programmed cell death (PCD), aberrant autophagic regulation, endoplasmic reticulum dysfunction, and intracellular calcium (see review [108]). The intrinsic apoptosis machinery of *C. elegans* comprises CED-9, CED-4, and CED-3, which is conserved from nematode to vertebrates and stimulates a protective response to mitochondrial dysfunction. JNK-1 promotes the expression of EGL-1 and CED-13. EGL-1 is required for all apoptosis, but CED-13 is required for pro-longevity signaling through the intrinsic pathway, i.e., mitochondrial reactive oxygen species (mROS) signaling pathway [80, 107]. Several stress factors, such as low IIS, heat stress, mitochondrial stress, and oxidative stress, among others, have been found to simulate the autophagic degradation of mitochondria (mitophagy). Besides sharing crucial regulatory factors with the general autophagy pathways, stressful conditions, specific components are also recruited for mitochondrial degradation. Outer mitochondrial membrane kinase PINK1 and its recruitment the cytosolic E3 ubiquitin ligase PARK2/parkin (also on the organelle) as well as their downstream signaling mediator, DCT-1, participate in mitophagy induction, mitochondrial homoeostasis protection, and survival promotion under stress conditions [65]. Mutations in PINK1 and PARK2 result in recessive familial forms of human Parkinson’s disease and correlate with mitochondrial dysfunction in mouse models [13, 58]. Similar to DAF-16, the transcription of SKN-1, which plays a key regulator in mitochondrial signaling pathway, has an influence on longevity and stress responses as well as NGDs [12, 107]. SKN-1 activation can be triggered by increased numbers of damaged mitochondria as well as increased cytoplasmic calcium levels upon mitophagy inhibition [65]. However, phosphorylation of SKN-1 by GSK-3 and kinases downstream from the *DAF-2* insulin-like pathway (AKT-1, AKT-2, and SGK-1) negatively regulate SKN-1 nuclear accumulation and activation [12, 98]. Prohibitin PHB complex, PHB1/PHB2, affect mitochondrial metabolism by sensing free radicals and/or mediating ROS production to influence longevity [3]. Interestingly, another negative regulator of SNK-1, WDR-23, has been found to interact with and regulate nuclear accumulation of SKN-1 but function independently of DAF-16 [12, 89].
Furthermore, Ca2+ released from the endoplasmic reticulum (ER) can also lead to ROS accumulation. The perturbation of ER Ca2+ homeostasis causes mitochondrial dysfunction, activating the mitochondrial-mediated apoptotic pathway, which has also been implicated in neuronal death in AD mouse model [59, 65]. Additionally, protein-folding stress at the ER stimulates the unfolded protein response (UPR), which is involved in the pathogenesis of many human diseases. For example, the basal activity of the UPR is beneficial under normal conditions but accelerates the pathology caused by toxic Ab protein in a *C. elegans* model of AD [74].

Genetic variants therefore produce a broad spectrum of biological effects. This can be seen in analysis of natural variants affecting late-onset AD (LOAD), where more than 20 genes involved in a range of processes including metabolism, inflammation, synaptic activity, and intracellular trafficking have been identified [45]. CNVs are also associated with variation in NGD phenotypes, with, for example, a rare duplication of the amyloid-β protein precursor linked to early-onset AD [35]. Thus, the question of how genetic variants affect mechanisms in diseases such as AD and PD remains challenging. Here, model species like the nematode *C. elegans* might offer a solution and provide more insight into the mechanism(s) underlying individual variation in NGD disease phenotypes.

### Natural variation associated with complex traits in *C. elegans* NGD models

*C. elegans* is a globally distributed nematode, and its level of natural genetic diversity is similar to human genetic variation [18]. Studying NGDs in a genetically tractable model species, such as *C. elegans*, allows detailed insight into the molecular pathogenesis. *C. elegans* models of AD, PD, HD, and other several NGDs have been established [22, 30, 31, 50, 84]. These models involve the transgenic expression of human genes under the control of a *C. elegans* promotor, with the resulting protein often linked to GFP or YFP. Applying fluorescent proteins (e.g., YFP) allows tracking of target proteins over time and the visualization of aggregation in vivo via the observation of the fluorescent foci (e.g., [101]). Such studies of AD using transgenic *C. elegans* have been carried out for many years. More recently, McColl et al. [53] have generated an improved model with human DA-Aβ 1–42 under the control of the muscle-specific promoter *unc-54* promotor. In this line, the full-length Aβ1-42 (the predominant Aβ species in human brain) is expressed in worm body wall muscle cells, and its oligomerizations and aggregations develop and result in severe. This fully penetrant, age progressive paralysis also shows more rapid than that caused from Aβ3-42 expression [53].

These transgenic models have all been generated in the N2 strain of *C. elegans* and have been widely used in screens for gene function analysis. However, such analyses in a single background have constrained the analysis and detection of natural allelic variants associated with complex traits. The most striking demonstration of this is a comparison of the RNAi phenotypes of ~1400 genes between N2 and CB4856 that showed that ~20% of genes differ in the severity of phenotypes between just these two genetic backgrounds [103]. Crucially, the natural variation found in *C. elegans* is also sufficient to cause significant changes in signaling pathways both at the gene expression (transcript and protein abundance) and phenotypic levels [87]. Accordingly, these could contribute to understanding how allelic variation affects gene expression, at the level of translation, in the multiple pathways and/or networks of complex traits involved in development and disease progression. For comparative investigations on mammalian genetics (including metabolism, aging, cancer, and neurodegeneration diseases), we therefore suggest that taking the different genetic backgrounds of the worm into consideration could be valuable. This approach would be likely to clarify proteostasis mechanisms and more fully reveal the relationships between genotype and phenotype. For example, in *Drosophila*, Chow et al. [14] showed 114 lines from the sequenced *Drosophila* Genetic Reference Panel of wild-derived inbred strains that exhibit the high heterogeneity in survival under endoplasmic reticulum (ER) stress conditions, and showed that 17 of 25 tested candidate genes were active in the putative response to ER stress [14].

Genetic and phenotypic differences between *C. elegans* populations have been identified with many traits shown to be variable between isolates (e.g., [2, 33, 40, 42, 90, 102]; for review, see [67]). Next to the canonical wild type Bristol N2, the most extensively characterized *C. elegans* isolate is the Hawaiian strain CB4856. This isolate is extensively diverged from N2, with a recent de novo assembly of the CB4856 genome suggesting that many variations have been maintained by balancing selection over long evolutionary timescales [94]. Wild *C. elegans* populations have also been shown to display significant local adaptation to their environment at multiple levels from the genotype and transcriptome [102]. Given this genetic and phenotypic variation, it is likely that variable natural genetic backgrounds of *C. elegans* will harbor abundant genetic variation that will modify the severity of NGD phenotypes. Wider analysis of more recently isolated wild isolates is also important as recent research has revealed specific adaptations to laboratory conditions that exist in the canonical wild-type Bristol N2 [5, 54, 66].

Studies into genetic background effects on NGDs in *C. elegans* have focused on a polyglutamine model. Here, Gidalevitz et al. [27] looked at the effects of natural genetic variation on susceptibility to aggregation and to toxicity [27]. This work introgressed a polyQ40 transgene from an N2 background into three wild genetic backgrounds, including the California-derived isolate DR1350, the Madeira isolate JU258, and the genetically distant Hawaiian isolate CB4856. A series of markers of pathology progression, such as onset of toxicity aggregation and the differential cell-specific susceptibility to aggregation, showed wide variation among the new introgression lines and between polyQ40 carrying recombinant inbred lines (RILs) derived from DR1350 and N2. This indicates that natural variation in genetic background can control resistance to misfolded protein aggregations and act to bind its associated cellular dysfunctions, which encourages further dissection on natural genetic variation susceptibility to age-related protein homeostasis in disease mechanisms.

Li et al. [49] mapped a large fraction of the *C. elegans* protein-protein interaction network. This was extended when the initial version of a human interactome map came out, adding more than 300 new connections to over 100 disease-associated proteins, including proteins related to NGD [73]. Lejeune et al. [48] accomplished a large-scale RNA interference screen in *C. elegans* strains that express N-terminal huntingtin in touch receptor neurons. Then, a subset of high-confidence modifier genes in pathways of interest in HD was identified by network-based analysis, including metabolic, neurodevelopmental, and pro-survival pathways. These results and those of similar large-scale analyses support the investigation of human disease pathways using *C. elegans* as a model (Table 1).

*C. elegans* offers the opportunity to investigate the genetic background modifiers affecting complex disease pathways (Table 2). For example, comparisons of RAS/MAPK signaling—a pathway critical in many complex human diseases, including AD [43]—across two different genetic backgrounds, N2 and CB4856, as well as their derived recombinant inbred lines identified the polymorphic monoamine oxidase *amx-2* (MAOA) as a negative regulator of RAS/MARK [77]. It was found that MAOA’s effects on RAS/MAPK signaling are produced by its effects on the level of the serotoninmetabolite 5 hydroxyindoleacetic acid (5-HIAA), the first endogenous small molecule identified to act as a systemic inhibitor of RAS/MAPK signaling [77].

**Table 2:** Quantitative genetic studies of genomic and phenotypic variation

The majority of quantitative trait locus (QTL) mapping approaches require inbred strains that have different alleles at loci affecting variation in the trait of interest and a polymorphic molecular marker linkage map. A cross between two genetically distinct parental strains or a series of crosses among parental lines produces recombinant inbred lines (RILs) or other segregating populations, such as introgression lines (IL). In most cases, the final panel is repeatedly inbred to obtain isogenic, and thus genetically stable, lines. As a consequence of random recombination events, each RIL combines different parts of the two (or more) parental genomes. In most previous research on *C. elegans*, the Hawaiian CB4856 strain—a strain that is highly divergent from N2 at many loci—and N2 have been used to detect QTLs [7, 70]. Various N2xCB4856 RILs and ILs present variable transgressive traits, and large genetic and phenotypic differences for a wide range of traits including reproduction, growth, and gene expression.
QTL mapping has however a number of shortcomings. Since the location of identified QTLs is indicated only by looking at which markers give the greatest differences between genotype class averages, QTL effects are often poor estimates of the true allele(s) effect. Moreover, when QTLs are far from markers—as has often been the case in earlier work when the markers are widely spaced—the mapping resolution is low. Furthermore, the network of genes as well as by environmental factors can regulate the complex dynamic process, such as disease progressing. Therefore, the QTLs or nucleotides (QTNs) that underlie a complex dynamic trait are expected to be characterized. Despite functional mapping provides a useful quantitative and testable framework for assessing the interplay between gene actions or interactions and developmental changes [105], further investigations are still needed through its opportunity to study development in a comprehensive manner and to study the dynamic network of genes that determine the physiology of an individual organism over time.
For understanding genetic pathways and gene networks, mapping of gene expression levels as quantitative trait loci (called eQTLs) has also been increasingly used for determining how a given variant affects gene expression. Sequence variants in eQTLs affect the expression of a gene, while other QTLs impact on any given trait of interest except from the trait causing by gene expression [71]. Owing to available measurement of either traits or phenotypes, eQTLs have been identified by evaluating gene expression in panels of genetically distinct genotyped individuals [29, 71].

### Genetic networks in stress and aging leverage the detection of NGD modifiers

NGDs are associated with other complex diseases (e.g., cancers). In part, this is a consequence of the involvement of the cellular systems that deal with various types of stress. For instance, variation in the genes that regulate the brain’s molecular response to oxidative stress are associated with differential neural vulnerability to the damaging effects of amyloid-β [34]. Here, oxidative phosphorylation takes place within mitochondria, to meet the elevated energy demands of neurons (Figs. 1, 2, and 3). However, this can also accelerate the accumulated oxidative damage, which could trigger impaired mitochondrial energy production and upregulate oxidative phosphorylation causing further DNA damage and significant levels of neuronal apoptosis [21].

A large number of genome-wide association studies (GWAS) in humans have identified pathways modulating the rate of aging and simultaneously influencing multiple age-related diseases such as NGDs [68, 76]. These GWAS studies have been invaluable in identifying and characterizing genetic variants associated with variation in disease phenotypes. For example, Seshadri et al. [83] identified 38 SNPs within 10 loci by integrating data from multiple studies and also identified CLU, PICALM, and CR1 as novel genes for late-onset AD (LOAD). A similar meta-analysis of GWAS studies of PD identified six more risk loci associated with the disease [60], while a combination of GWAS and whole-exome sequencing (WES) identified variants in the CEL gene/locus associated with PD [86]. Similarly, N’Songo et al. [61] identified ABCA7 missense variants that play a role in conferring AD risk in African Americans by performing WES with previously identified AD GWAS loci. According to their highlighted allelic heterogeneity at this locus, they also suggested the presence of additional AD risk variants in MS4A6A, PTK2B, and ZCWPW1.

NGDs are also strongly associated with aging, indicating that there is a link between protein misfolding and aging. This is very clear when the ILS (FOXO) pathway, a representative aging-related pathway, is considered. In *C. elegans*, this pathway ultimately controls the localization of DAF-16—a FOXO transcription factor that regulates a large number of genes involved in abiotic and biotic stress resistance, metabolism, and longevity—with dephosphorylation of DAF-16 allowing it to enter the nucleus [32]. The phosphorylation of DAF-16 is controlled by the insulin/IGF-1 transmembrane receptor ortholog *DAF-2*, with reductions in *daf-2* activity resulting in *daf-16*-dependent increases in lifespan. Loss of function *daf-2* mutations also increase lifespan in PD mutants of *C. elegans*, delaying the accumulation of small aggregates of alpha-synuclein in the body wall muscle and rescuing deficiencies in resistance to different stresses [17]. In humans, the age of onset of HD has a strong association with the length of polyQ expansion in the huntingtin protein but also varies between individuals with the same repeat length. In laboratory mouse strains, different genetic backgrounds can induce differential somatic expansion of CAG repeat.

### Perspectives: what is the genetic architecture of variation in NGD responses in *C. elegans*?

*C. elegans* quantitative genetics (Table 2) has yielded considerable insights into understanding complex human disease pathways, but there has been limited work in extending this to the analysis of NGDs. To this end, powerful and serviceable datasets relating to causal genetic variant exploration based on genomic analysis have been collected and can be obtained from WormQTL^HD^ (human disease) www.WormQTL-HD.org [100]. WormQTL^HD^ allows for systematically investigating phenotypic expression of *C. elegans* at levels equivalent to those of human diseases, by catalyzing integration of reported disease candidate gene associations, gene orthologue data, molecular profiles, phenotypic variations, and QTL results [93, 100]. Thus, this will support relevant available meta-datasets for human-worm studies and database exploration.

Taken together, *C. elegans,* a powerful model organism, can be used to study how variation affects the onset and progression of protein-misfolding disease and how the susceptibility to proteotoxicity performs in genetically diverse but phenotypically general individuals. This may explain the higher propensity to aggregation of the mutant disease-related proteins, in order to uncovering onset and phenotypes of disease patterns [27]. Regardless of the toxicity and aggregation of misfolded proteins, other factors leading to the variable complex traits could possibly include multiple additive or allele interactions, with the consequence of underlying intervention strategies at onset and/or in progression of disease. In addition, Paaby et al. [64] revealed cryptic genetic variations (CGVs) in the gene networks of *C. elegans* embryogenesis. CGVs are silent alleles in general and can be only activated to influence phenotype, when other functional genes are perturbed. The seemingly omnipresent cryptic-effect loci segregate at intermediate frequencies in the wild. However, they reveal low developmental pleiotropy. In the specific perturbations, e.g., changes of the molecular, cellular, or developmental processes that govern its phenotypic expression in complex metabolic human diseases, CGVs are required to be revealed [42, 56]; for reviews, see [25, 26]. Above all, the natural variation play an important role in neurodegeneration, involving inherently plastic genetic and molecular pathways, and might allow for description of complex etiology and implementation in eluding the harmful influences.

## Conclusions

Given the molecular conservation in neuronal signaling between *C. elegans* and vertebrates, including humans, this nematode is a valuable model species for studying NGD pathways and the alleles that affect them. Despite progress in identification of several AD- and PD-related genes, the effect of natural alleles underlying protein misfolding in these diseases remains mostly unclear. Here, we reviewed how natural variation could influence the severity of disease phenotypes. Experiments that rely solely on induced mutants in Bristol N2 limit the ability to explore how naturally varying alleles alter signaling pathways. Thus, further research should go beyond classic mutant screens on the genetic pathway analysis of complex traits, i.e., phenotypic differences among individuals, to also consider the allelic interactions in different genetic backgrounds. Consequently, the predictive nematode models of human genetic diseases could provide a more complete genetic and molecular understanding of how genetic variation shapes gene expression and cell biology for personalized genomic medicine.

## Abbreviations

5-HIAA: Serotoninmetabolite 5 hydroxyindoleacetic acid
AD: Alzheimer’s disease
ALS: Amyotrophic lateral sclerosis
APP: Amyloid precursor protein
Aβ: Beta amyloid peptide
BDNF: Brain-derived neurotrophic factor
CGVs: Cryptic genetic variations
CNV: Copy number variants
eQTLs: Expression quantitative trait loci
ER: Endoplasmic reticulum
FOXO: Forkhead box O protein
FTD: Frontotemporal dementia
GWAS: Genome wide association studies
HD: Huntington’s disease
HTT: Huntingtin
IIS: Insulin/insulin-like growth factor
ILs: Introgression lines
JNK: c-Jun N-terminal kinase
LOAD: Late-onset Alzheimer’s disease
LRRK2: Leucine-rich repeat kinase 2
MAOA: Monogeamine oxidase *amx-2*
MAPK: Mitogen-activated protein kinases
MLH1: MutL homolog 1
mROS: Mitochondrial reactive oxygen species
mTOR: Mechanistic target of rapamycin
mTORC2: Mammalian TORC2
NGDs: Neurodegenerative diseases
PCD: Programmed cell death
PD: Parkinson’s disease
polyQ: Polyglutamine
PrD: Prion diseases
QTLs: Quantitative trait locus
QTNs: Quantitative trait nucleotides
RILs: Recombinant inbred lines
ROS: Reactive oxygen species
S6K: Subunit S6 kinase
SNP: Single nucleotide polymorphism
UPR: Unfolded protein response
UPS: Ubiquitin/proteasome system
WES: Whole-exome sequencing
